# Hybrid surgery for blunt aortic injury with rupture: a case report

**DOI:** 10.1186/s13019-022-02060-w

**Published:** 2022-12-09

**Authors:** Takaaki Maruhashi, Hideo Maruki, Toshiaki Mishima, Tadashi Kitamura, Yutaro Kurihara, Marina Oi, Yuichi Kataoka, Kagami Miyaji, Yasushi Asari

**Affiliations:** 1 Department of Emergency and Critical Care Medicine, Kitasato University School of Medicine, 1-15-1 Kitasato Minami-Ku, Sagamihara, Kanagawa 252-0375 Japan; 2 Department of Cardiovascular Surgery, Kitasato University School of Medicine, 1-15-1 Kitasato Minami-Ku, Sagamihara, Kanagawa 252-0375 Japan

**Keywords:** Blunt thoracic aortic injury, Thoracic endovascular aortic repair, Hybrid surgery

## Abstract

**Background:**

Blunt thoracic aortic injury is one of the most lethal traumatic injuries. Ruptured cases often result in cardiac arrest before arrival at the hospital, and survival is rare.

Case presentation: A female patient in her 30 s was struck by an automobile while she was walking across an intersection. She was in a state of shock when emergency services arrived and was in cardiac arrest shortly after arriving at the hospital. A left anterolateral thoracotomy revealed a massive hemothorax secondary to thoracic aortic rupture. In addition, the patient had multiple traumas, including maxillary, pelvic, and lumbar burst fractures. We attempted to directly suture the aortic lesion; however, the increasing blood pressure caused the suture to break. We used a thoracic stent graft while ensuring permissive hypotension. Her postoperative prognosis was positive, and she was transferred to another hospital 85 days later.

**Conclusions:**

We successfully performed a hybrid surgery combining thoracotomy and endovascular repair for this emergency case of blunt thoracic aortic injury with rupture.

## Background

Blunt thoracic aortic injury (BTAI) is caused by high-energy trauma with horizontal and vertical deceleration mechanisms, and BTAI cases often involve multiple traumas with injuries in other parts of the body [1]. BTAI is the second most common cause of death in traffic accidents after head trauma, and only about 20% of cases reach the hospital before cardiac arrest [2]. Specifically, BTAI with mediastinal rupture usually causes cardiac arrest at the scene, and there have been few reports of survival. Thus, no treatment strategy has yet been established. We treated a patient who survived severe multiple traumas, including a ruptured BTAI that resulted in cardiac arrest immediately upon arrival at our hospital.

### Case presentation

**Fig. 1:**
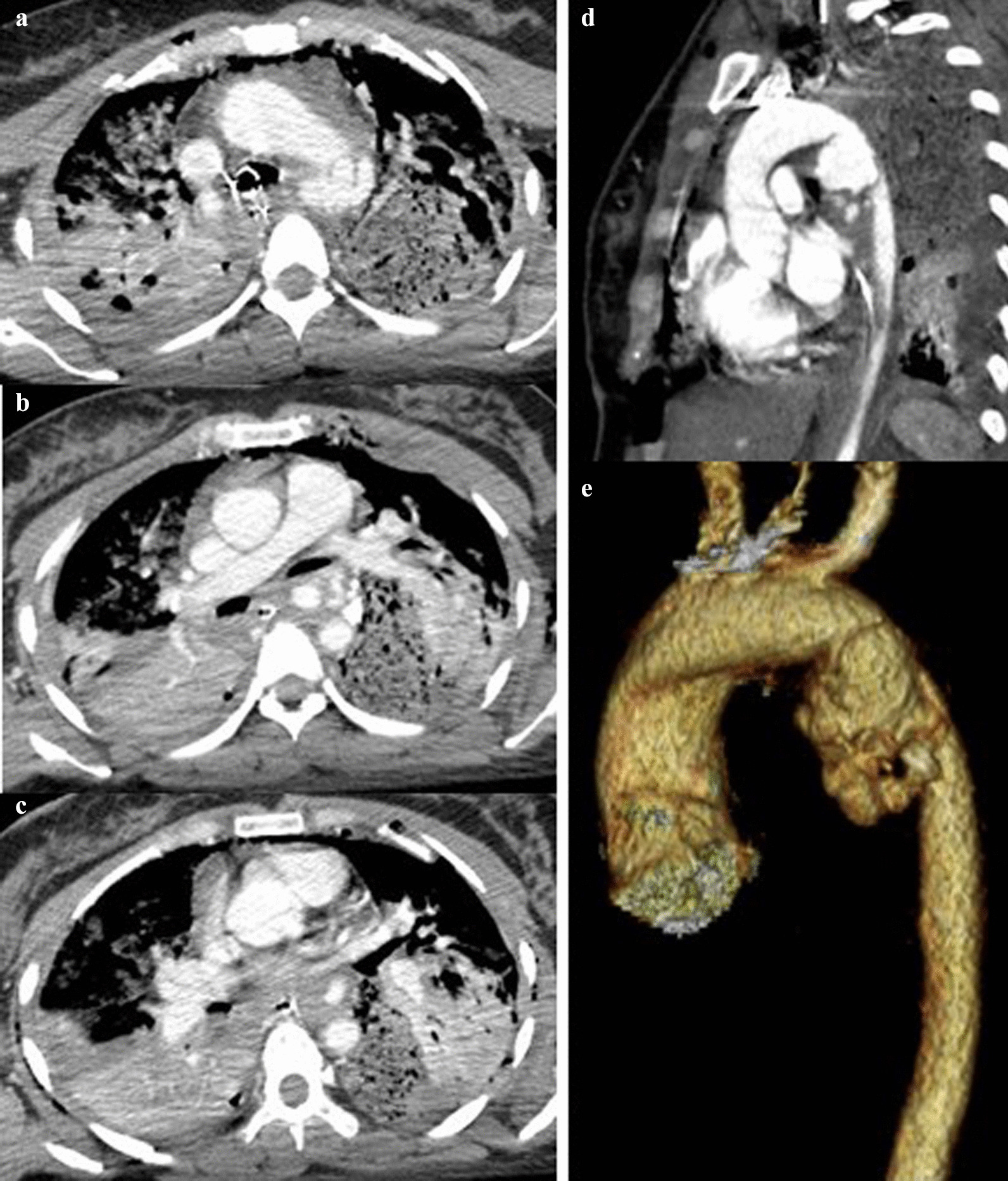
Preoperative contrast enhanced computed tomography of thoracic aortic injury. **a**–**c** Axial views, **d** Sagital view, **e** 3D Multiplanar reconstruction of aorta. Contrast enhanced computed tomography shows the aortic injury of isthmus and extravasation of contrast media into the mediastinum. Massive hemothorax was also observed in the bilateral thoracic cavity

A female patient in her 30 s had no relevant medical history or medication. When crossing the street, she was hit by an automobile and thrown approximately 5 m. On arrival at the hospital, her Glasgow coma scale was 13 points (E4, V4, and M5); respiratory rate, 30 breaths/min; SpO_2_, 100% (oxygen 10 L/min); blood pressure, 86/68 mmHg; and pulse, 142 beats/min. Subcutaneous emphysema and breath sound attenuation in the bilateral chest were observed. Tracheal intubation was performed immediately after bilateral chest drainage, and massive transfusion began. During these procedures, she went into cardiac arrest. We immediately performed a resuscitative left-sided thoracotomy and attempted aortic cross-clamping. There was a massive hemothorax of approximately 1000 mL in the left pleural cavity. No cardiac injuries were observed. Insertion of an aortic balloon occlusion (ABO) catheter from the right femoral artery was attempted at the same time as the thoracotomy; however, balloon inflation was interrupted because of suspicion of BTAI. Two minutes after resuscitation, spontaneous circulation returned. No active bleeding in the thoracic cavities was observed at that time. Computed tomography (CT) revealed a traumatic dissection in the aortic isthmus, which had partially ruptured and leaked contrast into the thoracic cavity (Fig. 1). Other injuries included a maxillary fracture, bilateral hemopneumothorax, multiple rib fractures, pelvic fracture with retroperitoneal hemorrhage, and a burst fracture of the first lumbar vertebra.

**Fig. 2:**
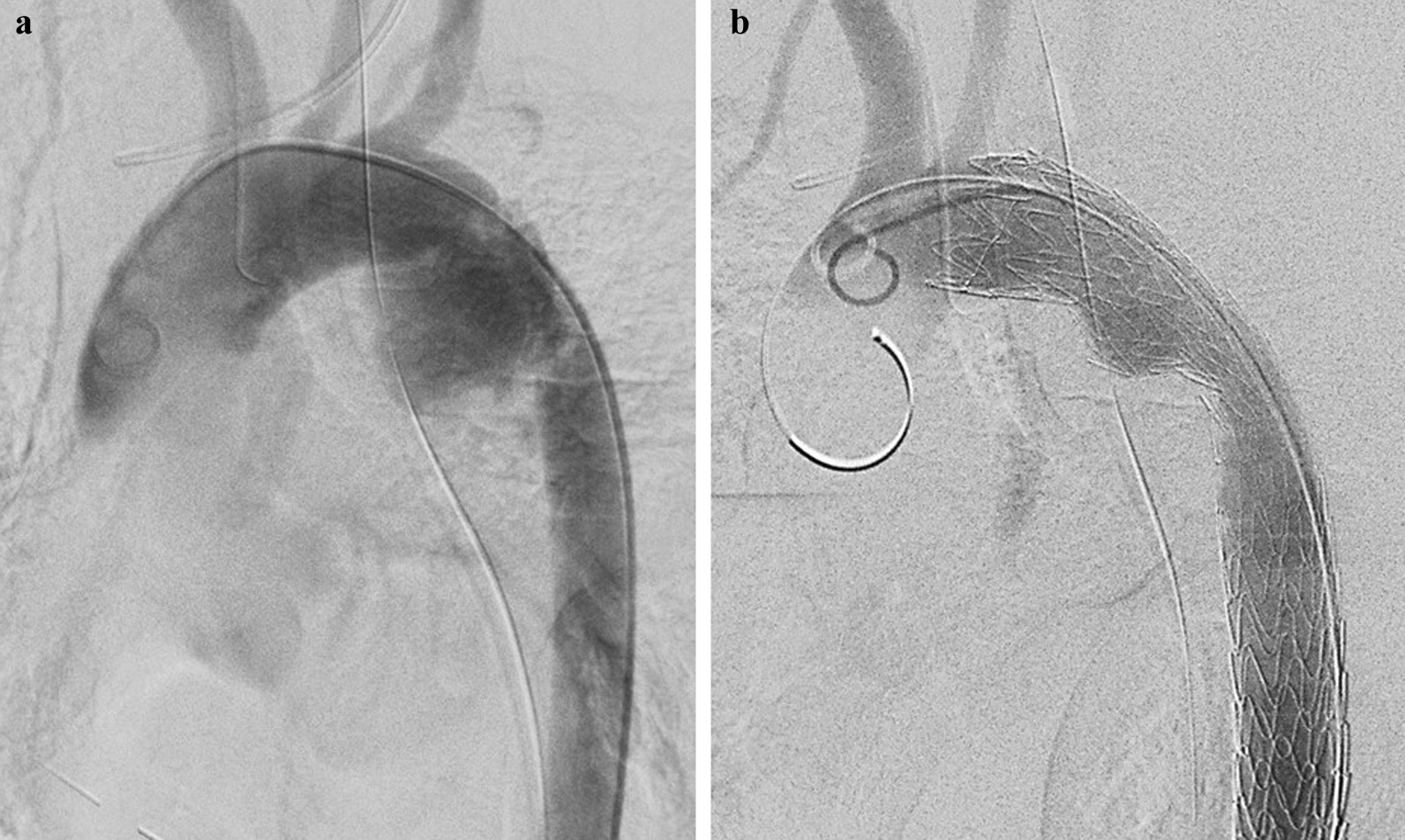
Angiogram during the thoracic endovascular aortic repair for traumatic aortic injury of the isthmus. **a** The aortic isthmus was irregularly dilated, and contrast extravasation was confirmed at this site. Intraoperative aortography imaging shows no extravasation of the contrast agent, indicating that temporary hemostasis was attained. **b** The proximal end of the stent graft is placed in the zone 2. No obvious endoleak of contrast agent was observed after stent graft placement

CT images showed reaccumulation of the left hemothorax, and the chest was reopened for hemostasis. Arterial hemorrhage (observed at the initial chest opening) was erupting into the thoracic cavity from the mediastinal lateral pleural tear. We concluded that the intrapleural rupture of the aortic injury was the cause of cardiac arrest. At initial presentation, the bleeding was considered to be temporarily hemostatic because the patient was in cardiac arrest. Temporary hemorrhage was controlled by exerting pressure on the vessel rupture point using the index finger, and direct suture of the injured area was attempted with horizontal mattress sutures using a polytetrafluoroethylene sheet. Suturing was difficult because the hemorrhage obscured visualization and the injured area had a partial aortic dissection. Although the bleeding appeared to temporarily stop, the false lumen at the injured site expanded with increased blood pressure, and the sutured area collapsed again, resulting in massive bleeding. We repeatedly performed direct suturing of the failed site while controlling the blood transfusion flow and managing the circulation with a systolic blood pressure of ≤ 80 mmHg. After temporary hemostasis was achieved, the patient underwent thoracic endovascular aortic repair (TEVAR) with a stent graft (GORE^®^ TAG^®^ 26 mm × 15 cm, W. L. Gore & Associates., Newark, DE, USA). The stent was located in zone 2 [3] (Fig. 2). Aortography showed no endoleaks, and blood flow in the left subclavian artery was maintained by retrograde blood flow from the left vertebral artery. After TEVAR and external fixation of the pelvic fracture, the patient was admitted to the intensive care unit.

The patient’s postoperative course was uneventful. The bilateral thoracic drains were removed on day 5, and extubation was performed on day 12. There were no neurological sequelae due to cardiac arrest, and follow-up CT showed no endoleaks or rebleeding. She was transferred to a rehabilitation hospital on postoperative day 85.

## Discussion and conclusions

The severity of BTAI is classified according to the American Society for Vascular Surgery. Grade I is aortic intimal tear only, and grade IV is aortic rupture. Grade II or higher is considered an indication for emergency surgery within 24 h [4]. Grade IV is divided into two categories: those with bleeding confined to the mediastinum and those with bleeding outside the mediastinum within the thoracic cavity, such as in this case. In a retrospective review of 332 BTAI cases treated at a Level I trauma center, Gerald et al. [5] reported eight grade IV cases (2.4%), but details such as the presence or absence of extra-mediastinal rupture were not described. Two of the 8 patients died shortly after arrival at the hospital before treatment, 3 of the 6 patients who were treated underwent surgical repair, and 3 were selected for TEVAR. Of these, one patient died after TEVAR because of uncontrolled bleeding after the procedure. However, the median time from hospital arrival to the start of surgery in the three cases in which surgical repair was performed was 4.0 h, and the reason for this long delay before radical treatment of a BTAI that had ruptured outside the mediastinum was not described. For BTAI rupture in the thoracic cavity with imminent cardiac arrest, as in the present case, it may be useful to perform prompt thoracotomy and temporarily suture the injured vessel on the thoracic side to control bleeding, followed by a hybrid procedure using TEVAR for hemostasis.

In recent years, resuscitative endovascular balloon occlusion of the aorta (REBOA) using an ABO catheter has rapidly become popular because it is a minimally invasive procedure, replacing aortic cross-clamping, which requires thoracotomy [6]. However, REBOA reportedly has the potential to increase bleeding proximal to the occlusion site [7, 8], making its application extremely limited in BTAI, which occurs mostly in the aortic isthmus. Therefore, we believe that when there is a suspected aortic injury or multiple traumas and the patient is in such an urgent condition that survival until CT imaging is unlikely, a resuscitative thoracotomy should be chosen without hesitation, as in this case. Not only was a direct approach to the injured area possible with rapid thoracotomy, but complications associated with ABO catheter insertion and balloon occlusion of the aorta were avoided.

Furthermore, this case demonstrates the efficacy of permissive hypotension in trauma resuscitation before complete hemostasis [9]. The efficacy of permissive hypotension has been confirmed in several large clinical trials [10, 11]. However, most of these cases were stab wounds, and there is no clear evidence that this technique can be also applied to blunt trauma. In this case, bleeding from the injured aorta could not be confirmed because the patient was in cardiac arrest or severe shock after resuscitation after the initial thoracotomy, but active bleeding from the injured area was confirmed at the second chest opening after the CT scan. With strict management of permissive hypotension, TEVAR was completed safely. This finding suggests that permissive hypotension before complete hemostasis may be effective for blunt trauma.

In conclusion, hybrid surgery combining rapid resuscitative thoracotomy, direct suturing of the aortic lesion, and endovascular treatment was successful in saving the life of a patient with severe multiple traumas and a ruptured BTAI in the thoracic cavity.

## Data Availability

Data are available from the corresponding author upon reasonable request.

## Abbreviations

ABO: Aortic balloon occlusion
BTAI: Blunt thoracic aortic injury
CT: Computed tomography
REBOA: Resuscitative endovascular balloon occlusion of the aorta
TEVAR: Thoracic endovascular aortic repair
